# Evolution of Depression and Anxiety among Breast Cancer Patients: a prospective analysis using clinical, biological and genetic factors

**DOI:** 10.1192/j.eurpsy.2024.436

**Published:** 2024-08-27

**Authors:** R. Khoury, R. Hachem, P. Salameh, H. Sacre, M. Bedran, L. Rabbaa, G. Chahine, J. Kattan, F. Nasr, A. Hajj

**Affiliations:** ^1^Faculty of Pharmacy; ^2^Laboratoire de Pharmacologie, Pharmacie Clinique et Contrôle de Qualité des Médicaments, Saint Joseph University; ^3^INSPECT-LB (Institut National de Santé Publique, d’Épidémiologie Clinique et de Toxicologie-Liban), Beirut; ^4^School of Medicine, Lebanese American University, Byblos, Lebanon; ^5^Department of Primary Care and Population Health, University of Nicosia Medical School, Nicosia, Cyprus; ^6^Faculty of Pharmacy, Lebanese University, Hadat; ^7^Department of Hemato-Oncology, Hôtel-Dieu de France Hospital, Faculty of Medicine, Saint Joseph University of Beirut, Beirut, Lebanon; ^8^Faculty of Pharmacy, Université Laval; ^9^Oncology Division, CHU de Québec- Université Laval Research Center, Quebec, Canada

## Abstract

**Introduction:**

Numerous studies have explored the symptoms and course of depression and anxiety in breast cancer patients and identified various clinical, sociodemographic, and genetic factors associated with their evolution. Nevertheless, these studies have been limited in duration and have focused on specific time points during chemotherapy or post-treatment follow-up. Furthermore, these studies included patients receiving different treatment regimens and used different tools to assess symptoms.

**Objectives:**

To assess the prospective evolution of depression in breast cancer patients over eight consecutive chemotherapy cycles, taking into account sociodemographic, clinical, biological, and genetic factors.

**Methods:**

A prospective longitudinal study was conducted on 69 breast cancer patients treated with intravenous chemotherapy at the oncology outpatient unit of the Hôtel-Dieu de France hospital (2017-2019; Ethics: CEHDF1016). The Hospital Anxiety and Depression Scale (HADS) was used to evaluate anxiety and depression in patients. Genotyping was performed for several genes (*ABCB1, COMT*, *DRD2, OPRM1*, *CLOCK, CRY2*, *PER2*) using the Lightcycler^®^ 2.0 (Roche).

**Results:**

Univariate repeated measures analysis showed differences in the evolution of depression and anxiety over time. For depression, a polynomial linear contrast for HADS-D scores was noted from cycle 1 to cycle 8, with a significant increase in depression at cycles 7 and 8 compared with cycle 1 (p-value _cycle7_=0.004 and p-value_cycle8_=0.009; Figures 1 & 2). Repeated measures analysis for anxiety showed a decrease in anxiety scores between cycles 1 and 6 of chemotherapy, followed by an increase starting cycle 6 (a polynomial trend for contrasts) (p-value _cycle6 versus 1_=0.038; Figures 1 & 2). Multivariable analysis showed that higher anxiety and depression scores at baseline were both associated with higher depression and anxiety scores over time. Other clinical and genetic factors, including polymorphisms in the *OPRM1*, *PER2,* and *COMT* genes, were also significantly associated with higher depression and anxiety scores.

**Image:**

**Figure:**
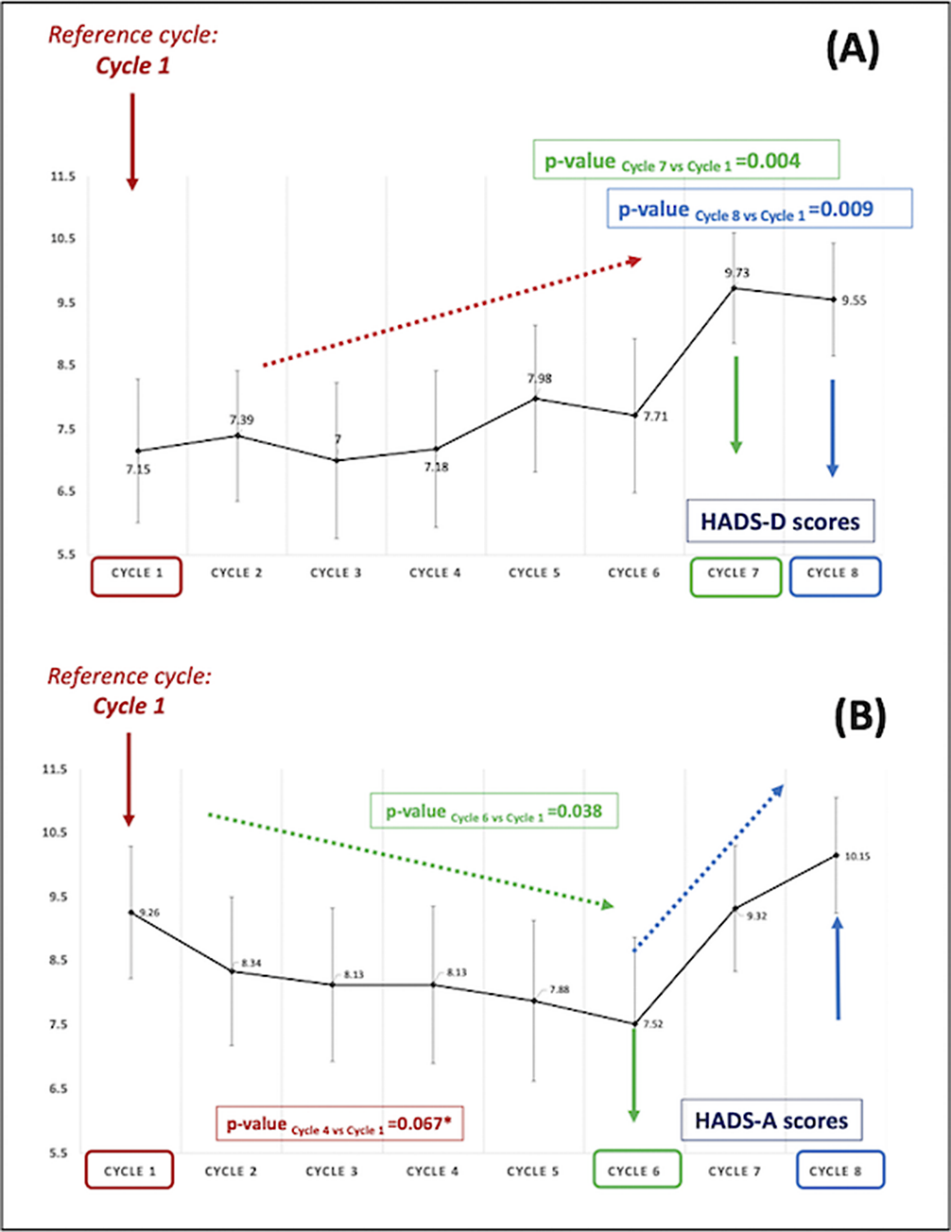

**Image 2:**

**Figure:**
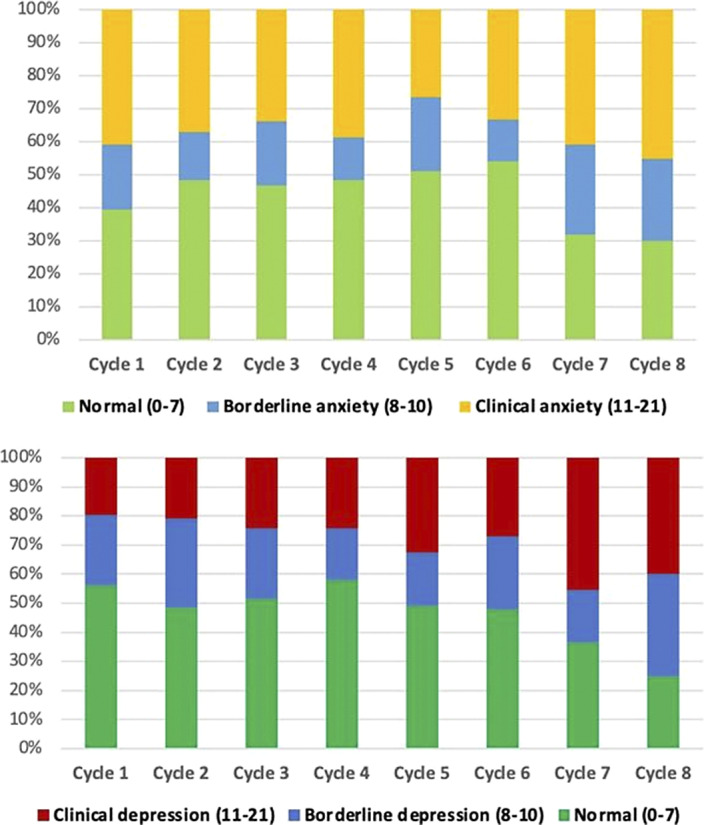

**Conclusions:**

Our findings highlight the importance of understanding the trajectories of depression and anxiety over time in women with breast cancer and identifying the triggering factors. Such personalized approaches would improve patient quality of life.

**Disclosure of Interest:**

None Declared

